# Reverse Cholesterol Transport: Molecular Mechanisms and the Non-medical Approach to Enhance HDL Cholesterol

**DOI:** 10.3389/fphys.2018.00526

**Published:** 2018-05-15

**Authors:** Leandro R. Marques, Tiego A. Diniz, Barbara M. Antunes, Fabrício E. Rossi, Erico C. Caperuto, Fábio S. Lira, Daniela C. Gonçalves

**Affiliations:** ^1^Exercise and Immunometabolism Research Group, Department of Physical Education, Universidade Estadual Paulista, Presidente Prudente, Brazil; ^2^Department of Cell and Developmental Biology, Institute of Biomedical Sciences, University of São Paulo, São Paulo, Brazil; ^3^Immunometabolism of Skeletal Muscle and Exercise Research Group, Department of Physical Education, Federal University of Piauí, Teresina, Brazil; ^4^Human Movement Laboratory, Universidade São Judas Tadeu, São Paulo, Brazil; ^5^Department of Biosciences, Universidade Federal de São Paulo, Santos, Brazil

**Keywords:** exercise, physical training, functional foods, nutritional strategies, lipoproteins, dyslipidemias

## Abstract

Dyslipidemia (high concentrations of LDL-c and low concentrations of HDL-c) is a major cause of cardiovascular events, which are the leading cause of death in the world. On the other hand, nutrition and regular exercise can be an interesting strategy to modulate lipid profile, acting as prevention or treatment, inhibiting the risk of diseases due to its anti-inflammatory and anti-atherogenic characteristics. Additionally, the possibility of controlling different training variables, such as type, intensity and recovery interval, can be used to maximize the benefits of exercise in promoting cardiovascular health. However, the mechanisms by which exercise and nutrients act in the regulation of cholesterol and its fractions, such as reverse cholesterol transport, receptors and transcription factors involved, such as PPARs and their role related to exercise, deserve further discussion. Therefore, the objective of this review is to debate about non-medical approaches to increase HDL-c, such as nutritional and training strategies, and to discuss the central mechanisms involved in the modulation of lipid profile during exercise, as well as that can be controlled by physical trainers or sports specialists in attempt to maximize the benefits promoted by exercise. The search for papers was performed in the databases: Medline (Pubmed), Science Direct, Scopus, Sport Discus, Web of Science, Scielo and Lilacs until February 2016.

## Introduction

Physical inactivity contributes to increased cardiovascular diseases, such as atherosclerosis, which are leading causes of death worldwide (Rosamond et al., 2008). The high concentration of low-density lipoprotein cholesterol (LDL-c) is an independent risk factor for coronary artery disease. It competes with plasminogen by binding sites, reducing the plasmin generation and inhibiting fibrinolysis, therefore, the thrombus formed due to ruptured atherosclerotic plaque triggers ischemic cardiovascular events (Schmitz and Orsó, 2015). On the other hand, there is an inverse relationship between high-density lipoprotein cholesterol (HDL-c) and incidence of coronary heart disease. It was shown that 1-mg/dL increment in HDL-c was associated with a significant decrease (3.7% in men and 4.7% in women) in cardiovascular disease mortality rates, according to a classical study conducted by The British Regional Heart (Gordon et al., 1989). In addition, recently, Madsen et al. (2017) showed that an extreme high concentration of HDL cholesterol was associated with all-cause mortality in both men and women. The authors shown that men with HDL cholesterol of 2.5–2.99 mmol/L (97–115 mg/dL) increased 1.36 (95% CI: 1.09–1.70) all-cause mortality and HDL cholesterol ≥ 3.0 mmol/L (116 mg/dL) were associated with a significant increase of 2.06 (1.44–2.95). For women, hazard ratios were 1.10 (0.83–1.46) for HDL cholesterol of 3.0–3.49 mmol/L (116–134 mg/dL) and 1.68 (1.09–2.58) for HDL cholesterol ≥3.5 mmol/L (135 mg/dL).

Several studies have investigated non-pharmacological strategies of prevention and treatment of dyslipidemia, such as enhanced HDL-c by increased physical activity, improved diet, or a combination of these in different populations. A recently systematic review analyzed the positives and negatives of behavioral counseling for the primary prevention of cardiovascular disease in adults without known cardiovascular risk factors. It was included 88 studies in this review (*N* = 121,190) in 145 publications and observed a reduction in the LDL-c (-2.58 mg/dL [95% CI, -4.30 to -0.85] in 13 trials [*n* = 5554]), and total cholesterol concentration (-2.85 mg/dL [95% CI, -4.95 to -0.75] in 19 trials [*n* = 9325]). They concluded that physical activity and behavioral diet programs for adults without high risk for cardiovascular disease showed benefits on LDL-c and total cholesterol. Additionally, the authors analyzed the dose-response effect of the interventions and found that higher-intensity exercise programs demonstrated greater improvements (Patnode et al., 2017).

Also, our group investigated the individual characteristics of body composition and metabolic profile that explain interindividual variation in HDL-c concentrations in response to 16 weeks of combined strength and aerobic training in postmenopausal women. We concluded that the positive responders had around 11% less HDL-c (6.31 mg/dL) at baseline than negative responders, suggesting that the positive response to combined training is also mediated by the metabolic health of the individual at baseline (Diniz et al., 2017).

Lipid metabolism involves several pathways that are, at least in part, interdependent, such as hepatic synthesis of very low-density lipoprotein (VLDL); uptake of fatty acids (FA) by skeletal muscle and / or adipose tissue; extrahepatic transport of cholesterol by low density lipoprotein; and removal of excess cholesterol by high density lipoproteins (HDL-c). Although exercise has been used as a cardioprotective intervention, the mechanisms involved still need to be fully elucidated. Therefore, the purpose of this review is to discuss the mechanisms involved in the HDL-c response during exercise, as well as how the manipulating of different training variables and nutrients could potentiate these benefits.

## Methods

Independent reviewers verified titles and abstracts and relevant full-text articles following specific inclusion criteria. Inclusion criteria for the present review were: 1- to assess lipoproteins; 2- to include exercise and nutrition intervention; 3- Adults and elderly; 4- obese and non-obese people; 5- smoking subjects; 6- with or without medication for cholesterol. The search for papers was performed using the following databases: Pubmed (Medline), Science Direct, Scopus, Sport Discus, Web of Science, Scielo, and Lilacs. The papers should be published between 2000 until February 2017, and the last examination was conducted in October 2017. We used the following keywords in English: physical exercise; aerobic exercise; strength exercise; nutritional approaches; supplements; lipoprotein; low density lipoprotein; high density lipoproteins, and lipoprotein metabolism.

## Lipoproteins

Lipoproteins are complex structures composed of lipids and proteins that transport lipids through body fluids. These particles are formed by a hydrophobic core containing triacylglycerol and esterified cholesterol, also containing on its surface a coating formed by a monophasic layer of phospholipids, non-esterified cholesterol, and apolipoproteins. Lipids and proteins composing plasma lipoproteins present different proportions, varying in size and density (Olson, 1998; Benoit et al., 2011; Feingold and Grunfeld, 2015).

The protein portion of lipoproteins is called apolipoproteins, which acts on the transport and metabolism of lipoproteins. These structures are important for maintaining the stability of lipoprotein structures because of its amphipathic characteristics, increasing solubility in aqueous environment, and are also important on activating and inhibiting enzymes of lipid metabolism. The most abundant apolipoproteins are A (apoA) and apolipoprotein B (apoB) (Shaeffer et al., 1978).

When the lipoproteins are in an aqueous environment, they go through a remodeling process, and their content can be carried between different classes of lipoproteins and different tissues. Dysfunctions in this process of remodeling during transportation may be related to different diseases, such as obesity, diabetes, coronary heart disease, and others (Sigal et al., 2011; Vinagre et al., 2013).

## Reverse Cholesterol Transport (RCT)

Reverse cholesterol transport is a mechanism by which the body removes excess cholesterol from peripheral tissues and delivers them to the liver, where it will be redistributed to other tissues or removed from the body by the gallbladder. The main lipoprotein involved in this process is the HDL-c. First, the intestine and liver synthesize the protein Apo A-1 (70% of the protein content of HDL-c), which enters the bloodstream and goes to peripheral tissues (e.g., heart). In veins and arteries, Apo A-1 interacts with receptors in various cell types (hepatocyte, enterocytes, and macrophages) called ATP-Binding Cassette, Sub-Family A (ABC1), Member 1 (ABCA1) (Leaf, 2003; Chapman et al., 2010).

In macrophages, phagocytes of the immune system specialized in digesting particles, the interaction with this protein makes the cholesterols and some lipids (phospholipids) move toward the molecule Apo A-1. This process results in the formation of nascent HDL-c particles (pre-β HDL), which subsequently can interact with *Scavenger receptor class B member 1* (SR-B1) and *ATP-binding cassette, sub-family G, member 1* (ABCG1), with the purpose of incorporating more cholesterol, forming a mature molecule of HDL-c (α-HDL). These processes are catalyzed by the enzyme Lecithin-cholesterol acyltransferase (LCAT).

Subsequently, there are two ways where cholesterol is delivered to the liver: direct and indirect. In the first, mature molecules of HDL-c interact with SR-B1 in the liver, which allows the transfer of its cholesterol content. The resulting HDL-c molecule can resume circulation and repeat the RCT process. Indirectly, mature molecules of HDL-c transfer its cholesterol content to apolipoproteins B-100 (Apo B-100), especially to the low-density lipoprotein (LDL), in exchange for triacylglycerol molecules. This process is catalyzed by the enzyme cholesteryl ester transfer protein (CETP). Thus, these lipoproteins can be associated with their liver receptors and deliver their cholesterol content (Cavelier et al., 2006; Rader, 2006). It is worth mentioning that CETP also catalyzes the reverse transference, i.e., triacylglycerol from HDL-c in exchange for Apo B-100 cholesterol.

HDL cholesterol content in plasma seems to be crucial in both prevention and treatment of atherosclerotic diseases, since this molecule exerts anti-inflammatory functions as well as exerts positive effects on CRT. However, recent studies have suggested that not only HDL cholesterol amount, but functionality has a much greater potential in some diseases such as coronary artery disease (CAD), chronic renal disease (CKD), dyslipidemia, diabetes and chronic ischemic cardiomyopathy. HDL may be affected by myeloperoxidase (MPO), 15-lipoxygenase (15- LPO), symmetrical dimethylarginine (SDMA) and other possible markers of this dysfunction. These effects altered HDL, reducing the availability of endothelial nitrate oxide, leading to problems of endothelial repair, increasing proinflammatory activation, and leading to efflux of macrophages, interfering in its functionality. These findings are only in the beginning and further researches must be conducted to understand the implications of abnormal HDL in the pathogenesis of atherosclerotic cardiovascular diseases and its clinic aplication. (Xu et al., 2013; Rosenson et al., 2016).

## Influence of Training Variables on Lipoproteins and RCT

Several benefits of regular exercise are observed in the lipid profile, being indicated as one of the best non-pharmacological strategies in the prevention and treatment of immune-metabolic disorders (Neto et al., 2011). In this perspective, exercise is indicated as treatment of several metabolic diseases because of its effective anti-inflammatory and anti-atherogenic effect. However, training variables, such as physical fitness condition, training models (aerobic or strength), intensity, volume, and recovery intervals, can be controlled in an attempt to obtain great effectiveness of different training models in the RCT as described in **Table 1**.

**Table 1:** Clinical evidence of the effect of training strategies on metabolic and lipid profiles.

Author	Sample	Exercise Protocol	Results
Gupta et al., 1993	Professional Athletes (*N* = 11)	Cycling and running exercising at the rate of 5.185 ± 501 kcal/week.	Free cholesterol flow LCAT activity CETP activity ↓LDL-c ↓Apo-B
Vaisberg et al., 2012	Professional Athletes (*N* = 14)	Marathon race exercising at the rate of 80–100 km/week	HDL-c ApoA-1 Transfers of non-esterified cholesterol triacylglycerol IL-6 TNF-α
Olchawa et al., 2004	Professional Atheles (*N* = 25)	Endurance exercises (triathlon, biathlon, running, and swimming) tested at the “off season” period	VO_2max_ HDL-c ApoA-1 pre-β1-HDL LCAT activity Cholesterol efflux capacity
Butcher et al., 2008	Sedentary Adults (*N* = 34)	Low-intensity exercise program exercising by walking 3 times/week (10.000 steps/session) during 8 weeks	↓Total cholesterol HDL-c oxLDL PPAR-gamma expression oxLDL scavenger receptor CD36 LXR-α ABCA1 ABCG1
Lira et al., 2009	Healthy men (*N* = 6)	Acute high-intensity aerobic exercise (∼ 90% VO_2max_) performed at cycle ergometer	↓Total cholesterol ↓LDL-c
Elmer et al., 2016	Sedentary men (*N* = 12)	Compared high-intensity training [12 intervals at 1 min work (90–100 V_V O2max_) with 1 min active recovery (50% V_V O2max_)], and moderate aerobic endurance training (70–80% V_V O2max_) during 8 weeks	↓Triacylglycerol Total colesterol/HDL-c ratio improvement
Hellsten et al., 1996	Adult men (*N* = 11)	Intermittent sprint cycle training (sprints of 10-s duration, repeated 15 times with 50 s of rest between each sprint) during 7 weeks	Glutathione peroxidase Glutathione reductase Phosphofructokinase activity Creatine kinase Anaerobic capacity improvement
Leggate et al., 2010	Healthy men (*N* = 11)	Compared high-intensity training (ten 4-min intervals at a power output to elicit 85–90% of VO_2peak_) and moderate intensity (60% VO_2peak_) in acute session	IL-6 soluble receptor (sIL-6R) IL-6/sIL-6R complex
Ostrowski et al., 2000	Professional Athletes (*N* = 53)	Running (average time of 206 min)	IL-6 IL-1ra Creatine kinase
Casella-Filho et al., 2011	Metabolic Syndrome subjects (*N* = 30)	Moderate intensity exercise training (45 min per day at 60 rpm performed 3 times/week) during 3 month	↓Triacylglycerol Free cholesterol Cholesterol ester transfers to HDL
Panissa et al., 2016	Healthy Adults (*N* = 12)	Compared high-intensity intermittent all-out exercise (60 × 8-s bouts interspersed by 12-s passive recovery) and fixed high-intensity intermittent exercise (100% maximal aerobic speed, consisted of 1-min repetitions at 70 rpm separated by 1-min of passive recovery) in acute session	Triacylglycerol VLDL
Rossi et al., 2016	Postmenopausal women (*N* = 65)	Compared combined training (strength plus aerobic exercise) and aerobic training during 16 weeks	↓Body fat ↓Percentage body fat Free fat mass HDL-c
Rossi et al., 2016	Healthy Adults (*N* = 8)	Compared the recovery time (30 or 90 s) in acute session of exhaustive strength exercises (four sets of squats and four sets of horizontal bench press) performed at 90% of 1RM	↓LDL-c HDL-c

Gupta et al. (1993) suggested that athletes (cyclists and runners) present a greater response in RCT pathways when compared to sedentary peers, with an increase of free cholesterol flow for esterification and subsequent transfer to other lipoproteins, such as HDL-c. These findings are reinforced in a recent study conducted by Vaisberg et al. (2012), which demonstrated that transfer of lipids to HDL-c is higher in athletes performing high-intensity activities when compared to sedentary individuals. However, this mechanism remains inhibited during the exercise against the inflammatory response of the organism. Thus, the anti-atherogenic response mediated by changes in the lipid profile seems to vary according to the individual’s physical fitness level (Olchawa et al., 2004).

Regarding the type of exercise, Kelley et al. (2004) conducted a meta-analysis about the effects of aerobic exercise on the lipid and lipoprotein profile between 1955 and 2003. The authors concluded that this type of exercise is effective in reducing total cholesterol (-2%), LDL-c (-3%), triacylglycerol (-5%) and increasing HDL-c (+ 3%) in women over 18 years of age.

In this perspective, the positive relationship between aerobic exercise training and lipid profile regulation is associated with enzymatic modulation of Lecithin-cholesterol acyltransferase (L-CAT), which is involved in the esterification of cholesterol during the RCT. Also, cholesteryl ester transfer protein (CETP) can transfer cholesterol ester particles of HDL-c to other lipoproteins (Grandjean et al., 2000; Durstine et al., 2002), given that both enzymes are associated with RCT. Additionally, another complementary positive effect activated during aerobic exercise is the hydrolysis of the TAG from VLDL by Lipoprotein Lipase (LPL), resulting, at least in part, in the formation of HDL-c particles (Oscai et al., 1982).

Butcher et al. (2008) aimed to investigate the effects of low-intensity chronic aerobic exercise in the modulation of lipid metabolism and performance of gene transcription variables (PPAR-γ e LXRα) responsible for controlling RCT in the liver. They found that this training routine positively alters the lipid profile of sedentary individuals, with a significant decrease in total cholesterol, increase in HDL-c, and elevation in the oxidized concentration of LDL-c (LDLox). It results in the activation and positive regulation of PPAR-γ and LXRα, and, consequently, a beneficial modulation of CD36, ABCA1, and ABCG1, being the last two proteins family members of the ATP binding cassette transporters directly associated with the regulation of RCT. In the following year, Lira et al. (2009) demonstrated that acute high-intensity aerobic exercise (∼ 90% VO2max) performed by healthy men on a cycle ergometer was able to reduce plasma concentrations of total cholesterol and LDL-c immediately and 1 h after the effort when compared to resting values.

In a study conducted by Elmer et al. (2016), they compared the chronic effects of high-intensity training [12 intervals at 1 min work (90–100 V_V O2max_) with 1 min active recovery (50% V_V O2max_)], and moderate aerobic endurance training (70–80% V_V O2max_) on chronic inflammation and changes in the lipid profile. The authors concluded that both training models should be adopted to maximize the health outcomes by significant alterations upon triacylglyc, when compared to moderate intensity endurance training. This significant and positive alteration could be the differential of high-intensity exercise in mediating significant responses on the lipid and inflammatory profile (Hellsten et al., 1996; Ostrowski et al., 2000; Leggate et al., 2010).

When analyzed subjects with an inflammatory profile established by non-transmissible chronic diseases, as metabolic syndrome, Casella-Filho et al. (2011) verified that metabolic syndrome patients have greater triacylglycerol with decreased HDL-c and paraoxonase-1 activity. In turn, after 3 months of moderate intensity exercise training (45 min per day at 60 rpm performed 3 times/week) was observed reduction in the triacylglycerol without alteration on HDL-c and LDL-c; however, was verified compositional changes in the smallest HDL subfractions associated with increased free cholesterol and cholesterol ester transfers to HDL suggesting a positive modulation even though in short-term in functional aspects of the lipoproteins.

However, when comparing the effect of different high-intensity training programs, Panissa et al. (2016) analyzed the acute lipid profile responses mediated by two protocols of high-intensity intermittent training (all-out exercise and fixed high-intensity intermittent exercise). They concluded that both modes of exercise lead to no improvements in lipid metabolism. The authors hypothesized that the energy expenditure in the two high-intensity intermittent protocols was not able to induce changes in the lipoprotein concentrations, mainly in HDL-c levels, given that previous study suggested improvements in HDL-c concentrations in exercise sessions with energy expenditure higher than 1100 kcal (Lira et al., 2012).

Tambalis et al. (2008) performed a systematic review comparing the effects of aerobic, resistance and combined (aerobic + resistance) training on the lipid profile and lipoprotein responses and observed that high-intensity aerobic exercise seems to induce greater benefits for the increase of HDL-c, and emphasize that resistance or combined exercise are important for improving functional and cardiovascular fitness. However, the wide variety of protocols makes it difficult to get a conclusion regarding lipid profile.

In a recent study, Rossi et al. (2016) compared the effects of aerobic and combined training (after equalizing load) on the lipid profile of postmenopausal obese women and concluded that combined training boosted concentrations of HDL-c, but no statistically significant difference was found when compared to aerobic exercise.

In the same research group, Rossi et al. (2016) also verified the influence of recovery interval (30 vs. 90 s) after four sets of squats and four sets of horizontal bench press, performed with 90% of 1 repetition maximum (1-RM) until momentary fatigue in previously trained subjects. As results, they observed that LDL-c decreased and HDL-c increased in both conditions, but without significant differences, suggesting that both intervals can be used when the goal is to improve lipid profile. However, studies investigating the influence of recovery interval after strength training are still scarce.

It is important to emphasize that the mechanisms involved in exercise-mediated beneficial responses are distinct in animal and human models; in this context, the latter sample is more explored and, given the practical applicability, the reproduction of the models should be tested in various populations. Therefore, while exercise promotes significant and favorable metabolic modifications mediated by RCT, the possibility of controlling acute training variables can be used by practitioners, coaches and/or trainers to induce superior benefits on lipid profile. However, studies investigating the influence of these variables in a chronic way are needed; moreover, the large variety of training protocols, especially regarding strength training, make comparisons and conclusions about the findings challenging.

## Exercise and its Relationship with RCT: Focus in the Receptors

As mentioned in the topic about RCT, some receptors are extremely important for HDL (pre-β and α) to achieve the uptake of cholesterol from peripheral tissues, such as ABCA1, ABCG1, and SR-B1. However, although it is well known that acute exercise and aerobic training are effective in causing favorable modifications on the lipid profile, the mechanism related to this outcome is still unclear.

Several studies have investigated the effect of exercise training, especially aerobic, on the modulation of proteins involved in RCT as described in **Tables 2** and **3**. Ghanbari-Niaki et al. (2007) found, after 6 weeks of aerobic training at 65% of maximum oxygen uptake (VO_2_max), an increase of approximately 30% in hepatic expression of ABCA1, as well as in plasma concentrations of total HDL-c, pre-β, and LCAT in Wistar rats. Years later, Khabazian et al. (2009) found similar results with Wistar rats that performed 6 weeks of aerobic training, showing increased gene expression of hepatic ABCA1 when compared to non-exercise rats.

**Table 2:** Summary of the effects of training strategies on lipids profile and RCT pathway in animals.

Author	Sample	Experimental design	Outcomes	Conclusion
Ghanbari-Niaki et al., 2007	10 adult Wistar rats	Treadmill running at 25 m/min (0% grade) for 90 min/day, 5 days/week for 6 weeks.	↑Hepatic gene expression of ABCA1 ↑Plasma HDL total and pre-β ↑Plasma LCAT	Endurance training increase plasma HDL-C levels may result from higher liver ABAC1 expression, LCAT, pre-b-HDL as key elements in RCT process.
Khabazian et al., 2008	10 adult Wistar rats	Treadmill running at 26 m/min (0% grade) for 90 min/day, 5 days/week for 6 weeks.	↑Hepatic gene expression of ABCA1	Endurance training increase hepatic ABCA1.
Khabazian et al., 2009	20 adult Wistar rats	Treadmill running at 25 m/min (0% grade) for 60 min/day, 5 days/week for 12 weeks.	↑Hepatic and intestinal gene expression of ABCA1 ↑Plasma HDC, HDL2, Apo A-1, pre-β and HDL total ↑Plasma LCAT activity	Endurance training increase elevation in plasma HDL-C and HDL2-C concentrations, accompanied by higher plasma Apo A-1, pre-β HDL-C concentrations, LCAT activity and ABCA1 mRNA expressions in rat intestine and liver.
Zhang et al., 2011	52 adult OLETF rats	Swimming training 5 days/week for 12 weeks.	↑Hepatic gene expression of PPAR-α, CPT-1, CAT, and ABCA1 ↑hepatic protein contend of PPAR-α	Endurance training increase hepatic PPAR-α that is a contributory factor to the improve whole-body lipid metabolism in diabetic rats.
Ghanbari-Niaki et al., 2011	10 adult Wistar rats	Treadmill running at 25 m/min (0% grade) for 60 min/day, 5 days/week for 12 weeks.	↑Gastrocnemius gene expression of ABCA1 ↑Plasma Apo A-1 and pre-β HDL	Endurance training improve lipids profile and muscle ABCA1.
Ngo Sock et al., 2014	50 adult Sprague-Dawley rats	Treadmill running at 26 m/min (10% grade) for 60 min/day, 5 days/week for 8 weeks.	↑Hepatic gene expression of SR-B1 and ABCA1	Endurance training seems to improve hepatic gene expression related to RCT.

**Table 3:** Summary of the effects of training strategies on lipids profile and RCT pathway in humans.

Author	Sample	Experimental design	Outcomes	Conclusion
Hoang et al., 2008	30 adult men	Sample was dichotomized by physical activity level	Physically active men had: ↑Apo A-1 and pre-β HDL ↑Leukocyte gene expression of ABCA1	ABCA1 expression in human leukocytes is associated with physical activity levels.
Butcher et al., 2008	34 adult men and women	Subjects underwent an treadmill exercise program that consisted of walking 10,000 steps 3 days/week for 8 weeks	Training group: ↑Plasma HDL ↑Leukocyte gene expression of PPAR-γ, LXRα, FAT-CD36, ABCA1 and ABCG1 ↑PPAR-γ DNA-binding activity in leukocytes	Low intensity exercise improves serum lipids, as a result of increased PPAR-γ and LXRα and consequently up-regulation of CD36, ABCA1, and ABCG1 within circulating leukocytes.
Ghanbari-Niaki et al., 2011	20 adult women	Subjects performed a single session of circuit resistance exercise (9 exercises), in three different intensities (40, 60 and 80% of one-repetition maximum)	Intensity of 60 RM% ↓Plasma VLDL ↑Lymphocyte gene expression of ABCA1	A single session of circuit resistance exercise increased lymphocyte ABCA1 expression that was more pronounced in 60% RM.
Greene et al., 2012	16 obese adult men and women	Subjects underwent endurance training at 70% of VO_2max_ (∼500 kcal/session) 3 days/week for 12 weeks	Training group: ↑Muscle protein content of PPAR-α and δ ↑Muscle protein content of AMPKα, PGC-1α, FAT-CD36 and CPT-I ↔Muscle protein content of ABCA1	Endurance training enhanced expression of PPARs, PGC-1α and AMPKα that were related to improvement in lipids profile.

The positive effects of exercise on receptors related to RCT was also observed in longer experimental protocols. For example, Khabazian et al. (2009) found that Wistar rats, after 12 weeks of aerobic training at approximately 65% of VO_2_max, increased gene expression of ABCA1 in the liver and small intestine (also increased in the heart [Ghanbari-Niaki et al., 2013]). The authors also observed an exercise-mediated improvement of LCAT activity. When combined, these alterations resulted in higher HDL-c and its subfractions, as well as pre-β HDL and Apo A-1.

Exercise seems to improve RCT receptors in non-communicable diseases in experimental models. In mice with type 2 diabetes, physical training induced an increase of ABCA1 (Zhang et al., 2011). Additionally, Zhang et al. (2011) observed increased gene expression and protein content of PPAR-alpha in the liver, indicating, in parts, that this transcription factor could play an important role in exercise-mediated improvement in hepatic lipid metabolism.

Few studies have examined the effect of physical training on SR-B1 modulation. In mice genetically modified to develop atherosclerosis, Jinli and Jidi (2002) verified that 10 weeks of aerobic training induced an increase in gene expression and protein content of SR-B1, finding that was associated with a reduction of atherosclerotic plaque. Recently, Ngo Sock et al. (2014) showed an increased gene expression of SR-B1 and ABCA1 after 8 weeks of aerobic training in ovariectomized rats.

In humans, studies are mostly limited to the analysis of proteins involved in RCT through the culture of plasma blood mononuclear cells, such as monocytes and lymphocytes. Hoang et al. (2008) found that physically active individuals had higher concentrations of Apo A-1 (the main protein of HDL), mRNA of ABCA1 in leukocytes (but not in skeletal muscle) and pre-β HDL than sedentary individuals. Moreover, there was a positive correlation between ABCA1 in leukocytes with pre-β HDL and LCAT activity, as well as Apo A-1 and cholesterol efflux, indicating the importance and relationship of these proteins in RCT.

Interestingly, the results are similar after an acute session of exercise at 60% of maximal intensity, showing an improvement in the lipid profile mediated by the decrease of VLDL and the LDL/HDL ratio, as well as increased expression of ABCA1 in lymphocytes (Ghanbari-Niaki et al., 2011). Butcher et al. (2008) observed that even low-intensity training (10,000 steps three times a week) positively alters lipid profile in sedentary individuals, mediated by the decrease in total cholesterol and increase of HDL-c. In addition, exercise caused elevation of oxidized LDL-c concentrations, which resulted in the activation and positive regulation of PPAR-γ and LXRα transcription factors in leukocytes, and consequently, increase in gene expression of cholesterol-related reverse transporters to HDL-c, such as FAT-CD36, ABCA1, and ABCG1.

Trying to clarify the mechanisms by which acute and persistent exercise improve lipid profile and consequently decrease the risk of development of cardiovascular diseases, studies carried out in the 21st century investigated the role of PPAR as a possible regulator of lipid and glycemic metabolism in RCT. Incited by the study conducted by Repa et al. (2000), which observed that several nuclear receptors that formed heterodimers with the RXR complex, such as PPAR, had the capacity to regulate gene expression of ABCA1, Oliver et al. (2001) found that when macrophages, lymphocytes, and intestinal cells were treated with PPAR agonist, these cells increased ABCA1 gene expression and induced an increase of ApoA-1 cholesterol uptake. In addition, the PPAR agonist was effective in increasing HDL-c and decreasing LDL-c. However, the last study did not confirm this mechanism on the effect of exercise.

However, a recent study suggested that exercise-induced activation of PPAR, especially the delta isoform, may stimulate further transcription factors that are related to genes involved in RCT (Gervois et al., 2007; Greene et al., 2012). Greene et al. (2012) investigated the involvement of PPAR delta in the improvement of lipoproteins among obese individuals after 12 weeks of aerobic training. As results, they found that training increased 39 and 122%, respectively, of the protein content of PPAR alpha and delta in the muscle of trained individuals. Also, AMPK content increased 93% when compared to baseline in trained individuals. Similarly, there was an increase of FAT/CD36, lipoprotein lipase, CPT-1 and COX-IV in the trained individuals. However, the protein content of ABCA1 and LDLR did not change with exercise. Interestingly, increased AMPK was positively correlated with higher HDL-c concentration (total and subfractions), and the increase in PPAR delta was negatively correlated with LDL-c concentrations. These results suggest, in parts, that in the muscle the decrease of LDL-c and the increase of HDL-c may be dependent, respectively, of PPAR delta and AMPK. Additionally, Greene et al. (2012) did not find differences in the expression of ABCA1 in the muscle, corroborating with previous studies that showed an increase of mRNA of this protein only in leukocytes (Hoang et al., 2008) and in the liver (Khabazian et al., 2009).

**FIGURE 1:**
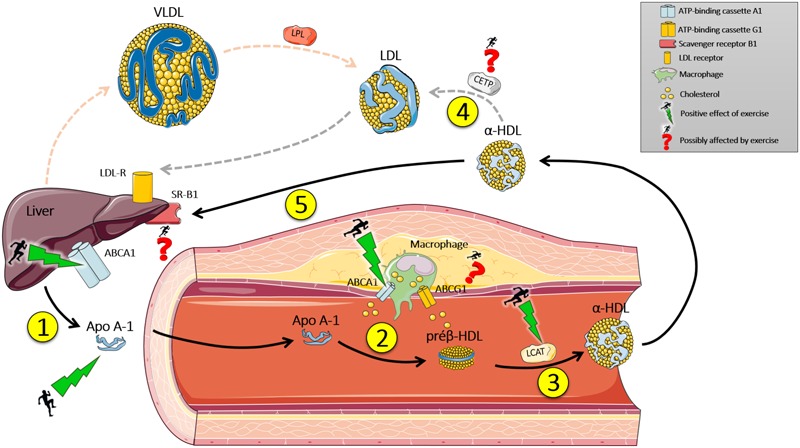
Effect of exercise on reverse cholesterol transport (RCT). (1) The liver synthesizes Apo A-1 that enters the bloodstream. (2) Apo A-1 has an affinity to ABCA1, present in mononuclear cells of the immune system (macrophages), which transport cholesterol from the peripheral tissues to Apo A-1, which becomes a pre β -HDL (discoidal). (3) Pre β – HDL binds to ABCG1 which transports cholesterol from the peripheral tissues to the molecule, which is modified by LCAT enzyme (transporting cholesterol from the surface of the pre β – HDL surface to the central part) becoming α-HDL (mature HDL-c). (4) α-HDL is modified by the enzyme CETP, which replaces the lipid content of LDL-c (triacylglycerols) by cholesterol from α-HDL, and then binds to its receptor in the liver (LDL-R) and deposits its cholesterol contents. (5) Or, α-HDL can return to the liver and transfer its cholesterol content to the liver through SR-B1. Next, cholesterol can be reused or expelled by the gallbladder. Exercise can modulate genetic and protein expression of Apo A-1, ABCA1 hepatic and immune system cells, and increase the activity of Lecithin-cholesterol acyltransferase (LCAT). Studies about the effect of exercise on cholesteryl ester transfer protein (CETP) activity, SR-B1, and ABCG1 expression are inconclusive. Black arrows indicate direct RCT. Dotted gray arrows indicate indirect RCT. Dotted red arrows indicate the hepatic secretion of VLDL, which is modified by lipoprotein lipase (LPL) in the bloodstream and hydrolyzes its lipid content to peripheral tissue and subsequently becomes LDL-c. Apo A-1, Apolipoprotein A-1.

In summary, physical training, especially aerobic exercise, increases the expression of AMPK and PGC1-alpha, which combined can activate PPAR. When activated, it moves to the nucleus and connects to its promoter region, where it transcribes genes related to oxidative metabolism and transport of lipoproteins, such as ABCA1 and Apo A-1. These outcomes may provide RCT, and consequently, a reduced risk of developing cardiovascular disease (**Figure 1**). Despite the importance of PPAR and other receptors during RCT, it is essential to mention that this transcription factor responds to exercise in several ways, including angiogenesis, inhibition of lipogenesis and mitochondrial activity, suggesting the need for future studies about the mechanisms of RCT, exercise and the role of PPAR.

## HDL-c and Nutrition

Many studies have demonstrated that diet and nutrition have a crucial role in the metabolism of lipoproteins, including HDL-c levels. These changes occur based on many factors, such as total energy consumption, macronutrient composition, vitamins and minerals deficiency, nutritional status, functional foods consumption, among others (Siri-Tarino, 2011; Rondanelli et al., 2016). However, the nutritional factors can contribute in different manners, depending on gender, age, genetic factors and presence of some pathologies.

Hypocaloric diets appear to increase HDL-c, as shown in a study performed by Loftus et al. (2015). The change was linked to a reduction in total carbohydrate intake. Many studies have reported the effects of low-carb diet to improve HDL-c levels (Siri-Tarino, 2011; Mooradian and Haas, 2014; Thorning et al., 2015). Regarding macronutrient composition, low-carb diets showed the best results in increasing HDL-c levels. A rich carbohydrate diet is associated with lower HDL-c levels, regardless the type of carbohydrate intake (complex carbohydrates, fructose or glucose) and glycaemic index (Siri-Tarino, 2011).

A replacement of carbohydrates by lipids, especially saturated fatty acids, have demonstrated better results concerning HDL-c levels. Thorning et al. (2015) showed that diets rich in cheese and meat increased HDL-c levels when compared to the same energy intake diets, but composed of carbohydrates instead of saturated fatty acids. Engel and Tholstrup (2015) showed that butter consumption could increase HDL-c levels when compared to olive oil. Monounsaturated and Polyunsaturated Fatty Acids also have beneficial effects on HDL-c levels, especially olive oil, that contains Monounsaturated Fatty Acids and polyphenols (Siri-Tarino, 2011; Chen et al., 2014; Farràs et al., 2015; Hernáez et al., 2016; Rondanelli et al., 2016). Trans fatty Acids are responsible for lowering HDL-c levels in humans.

Protein intake doesn’t seem to interfere in HDL-c levels (Mooradian and Haas, 2014). However, soy protein supplementation appears to increase HDL-c levels, but due to isoflavones intake (Ma et al., 2005; Rios et al., 2008; Palacio et al., 2009; Squadrito et al., 2013). Moderate alcohol consumption appears to increase HDL-c levels in humans (Mooradian and Haas, 2014).

Many functional foods and bioactive compounds are associated to improve HDL-c levels in animals and humans. Linseed oil (Avelino et al., 2015), cocoa (Basu et al., 2015), pistachios (Hernández-Alonso et al., 2015), artichoke leaf, red yeast rice, olive oil, and bergamot (Rondanelli et al., 2016) have been used as a strategy to increase HDL-c levels. Bioactive compounds, such as Astaxanthin (Kishimoto et al., 2016), lycopene (Palozza et al., 2012), Curcuma Zedoaria Roscoe (Tariq et al., 2016), mangiferin (Na et al., 2015), probiotics (Thushara et al., 2016), resveratrol, and anthocyanins (Mooradian and Haas, 2014) seem to be interesting options of treatment to raise HDL-c levels.

Although studies with functional foods and bioactive compounds still prove inconclusive, a specific group of bioactive compounds has shown promising results. Flavonoids are polyphenols that are dissolved in other classes, making up a group of more than 500 members. These compounds are present in fruits, and foods like teas, wine, chocolate, among others (Millar et al., 2017). The main flavonoids and their forms of action are described below.

### Anthocyanidins

This group is mainly composed of cyanidin, peonidin, pelargonidin, malvidin, delphinidin, and petunidin. They are found as pigments in fruits such as berries, petals and leaves. Animal studies demonstrate that anthocyanidins act in two different ways: their cardiovascular action is associated with their anti-inflammatory properties and stimulating the RCT (Wang et al., 2012).

### Flavanols

The subclass of flavanols is composed mainly of catechins, commonly found in cocoa, wine, grape juice and green tea. The action of the catechins is related to an increase in serum PON 1 activity and a decrease in ApoB lipoprotein oxidation in diabetic rats (Tas et al., 2005). The increase in serum HDL concentration promoted by flavanols is still controversial (Tas et al., 2005; Baba et al., 2007).

### Flavanones

This subclass of flavonoids is composed mainly of naringenin and hesperitin, present in citrus fruits, tomatoes and peels. Flavanones have shown decreased activity of PON 1, a major anti-atherosclerotic component of high-density lipoprotein (Kim et al., 2010). Another study performed with orange juice intake showed an increase in serum HDL in rats (Haidari et al., 2012).

### Isoflavones

Isoflavones are a group composed of daidzein and genistein, present mainly in soybean, and have a distinct composition from other flavonoids, since its chemical structure is similar to that of estrogen. clinical studies have shown that soy protein intake may only cause a slight increase in serum HDL cholesterol concentration (Weggemans and Trautwein, 2003), however, supplementation with isoflavones appears to have a significant improvement in postmenopausal women (Taku et al., 2007).

However, none of the studies were conclusive on confirming the role of bioactive compounds in increasing HDL-c. The isolated supplementation of these compounds or the use of functional foods does not seem to help to increase HDL-c levels since there is only weak evidence available. Therefore, the increase of HDL-c is not only associated with one factor but the combination of diet and physical exercise.

## Future Perspectives

Systematic exercise can cause epigenetic adaptations, among them, for example, the methylation of some genes related to lipid metabolism (Guay et al., 2013). A recent study found that methylation of lipoprotein lipase (LPL) in human leukocytes was positively correlated with HDL-c concentrations and its particle size. In both sexes, methylation of CETP was negatively correlated with LDL-c concentration. In men, CETP methylation was negatively correlated with concentration and molecule size of HDL-c and positively correlated with HDL-triacylglycerol (Guay et al., 2013). Nutrients are also related to HDL-c increase and its functionality. However, there are weak evidences that isolated supplementation of bioactive compounds are capable of increasing HDL-c.

Therefore, it may be suggested that the combination of specific nutrients, functional foods ingestion plus regular exercise can promote a greater activity of RCT, an increase of HDL-c and consequently, a better metabolic profile.

## Author Contributions

LM, FL, and DG conceptualized and designed the study and contributed to analysis and interpretation. TD, BA, and FR performed the data collection and drafting of the paper. EC revised the work and final approval of the manuscript.

## Conflict of Interest Statement

The authors declare that the research was conducted in the absence of any commercial or financial relationships that could be construed as a potential conflict of interest.
